# Pediatric Patient with Ischemic Stroke: Initial Approach and Early Management

**DOI:** 10.3390/children8080649

**Published:** 2021-07-28

**Authors:** Jozef Klučka, Eva Klabusayová, Tereza Musilová, Tereza Kramplová, Tamara Skříšovská, Milan Kratochvíl, Martina Kosinová, Ondřej Horák, Hana Ošlejšková, Petr Jabandžiev, Petr Štourač

**Affiliations:** 1 Department of Paediatric Anaesthesia and Intensive Care Medicine, University Hospital Brno and Faculty of Medicine, Masaryk University, Kamenice 5, 62500 Brno, Czech Republic; Klucka.Jozef@fnbrno.cz (J.K.); Klabusayova.Eva@fnbrno.cz (E.K.); Musilova.Tereza@fnbrno.cz (T.M.); Kramplova.Tereza@fnbrno.cz (T.K.); Skrisovska.Tamara@fnbrno.cz (T.S.); Kratochvil.Milan@fnbrno.cz (M.K.); Stourac.Petr@fnbrno.cz (P.Š.); 2 Department of Paediatric Neurology, University Hospital Brno and Faculty of Medicine, Masaryk University, Kamenice 5, 62500 Brno, Czech Republic; Horak.Ondrej@fnbrno.cz (O.H.); Oslejskova.Hana@fnbrno.cz (H.O.); 3 Department of Paediatrics, University Hospital Brno and Faculty of Medicine, Masaryk University, Kamenice 5, 62500 Brno, Czech Republic; Jabandziev.Petr@fnbrno.cz

**Keywords:** ischemic stroke, pediatric, child, management, initial approach

## Abstract

Acute Ischemic Stroke (AIS) in children is an acute neurologic emergency associated with significant morbidity and mortality. Although the incidence of AIS in pediatric patients is considerably lower than in adults, the overall cumulative negative impact of the quality of life could be even higher in children. The age-related variable clinical presentation could result in a delay in diagnosis and could negatively influence the overall outcome. The early management should be based on early recognition, acute transfer to pediatric AIS centre, standardised approach (ABCDE), early neurologic examination together with neuroimaging (preferable Magnetic Resonance Imaging—MRI). The treatment is based on supportive therapy (normoxemia, normocapnia, normotension and normoglycemia) in combination with intravenous/intraarterial thrombolytic therapy and/or mechanical thrombectomy in selected cases. Pediatric stroke centres, together with the implementation of local stroke management protocols, could further improve the outcome of pediatric patients with AIS.

## 1. Introduction

According to the World Health Organization (WHO), a stroke is defined as “rapidly developing clinical signs of focal (or global) disturbance of cerebral function, with symptoms lasting 24 h or longer or resulting in death, with no apparent cause other than of vascular origin” [1]. However, an updated definition of stroke for the 21st century could be currently preferred and written as follows: “an acute onset neurological sign or symptom attributable to focal brain infarction or haemorrhage” [2]. A stroke is an acute neurologic emergency with the need of urgent diagnosis, central nervous system imaging and prompt treatment, ideally in the set time window. A stroke in pediatric patients is associated with significant morbidity and mortality [3]. Acute Ischemic Stroke is among the top ten causes of death, with the greater risk in specific subpopulations (adolescents and infants, patients with sickle cell anaemia, black race and male gender) [4,5,6]. In comparison to adults, the incidence of acute stroke in pediatric patients is considerably lower. However, the proportion of patients with lasting neurologic deficit [7,8,9,10], the impact on quality of life and the health care system and overall cumulative care costs could be significantly higher in children [9,11,12]. This narrative review aims to primarily investigate AIS in pediatric patients.

## 2. Classification

When considering the aetiology of a stroke, there are two major subtypes of strokes: ischemic and hemorrhagic. Although the supportive therapy (neurointensive care) has the same groundings in both cases, primary treatments are entirely different (thrombolysis, thrombectomy, anticoagulation and antiplatelets in ischemic aetiology in contrast to coagulation management and surgical intervention in haemorrhagic stroke). Ischemic strokes can be caused by arterial or venous pathology. Venous-based strokes are caused by cerebral sinovenous thrombosis (CSVT) or by cortical vein thrombosis [13].

The age-related classification of AIS divides this clinical syndrome into two categories: perinatal stroke (sometimes referred to as neonatal) and childhood stroke [13]. Perinatal stroke is classified as a stroke during the perinatal period from 20 weeks (sometimes 28 weeks) of gestation until the end of the newborn period (28th postnatal day). Childhood stroke encompasses the age group from 28 days up to 18 years of age [14]. Silent stroke is defined only for clinical purposes as abnormal central nervous system or vascular system of head and neck abnormality without neurologic presentation and neurologic deficit; however, according to the definition—acute neurological symptoms [2]—this could not be defined as a stroke per se [13]. Nevertheless, the abnormalities found on radiology imaging can be associated with a slow cognitive impairment in adults [4,13]. Recurrent strokes are defined as a repeated stroke insult over time in the same patient. The incidence of recurrent stroke could reach up to 12% in the first year after the initial stroke insult [15], with the highest risk in patients with arteriopathy or cardiac diseases. The recurrence risk could reach up to 27% [16].

## 3. Epidemiology

The reported incidence of strokes in pediatric patients is between 1.3–13/100,000/year and up to 25–40/100,000/births in neonates [15,17,18,19] with an increasing trend over the past decades that is possibly due to the improvement in stroke detection and availability of imaging methods [20]. The overall incidence of stroke in childhood is age-related, with the peak in the perinatal period (up to 25% reported cases) [6,21,22] and progressively age-related decrease. In childhood strokes (excluding the perinatal period), the highest published incidence is below five years [23,24,25,26,27,28], with a median age of 2.3 years [29]. In the perinatal period, the dominant type of stroke is AIS with arterial ischemic aetiology (up to 80%) [13]. In childhood, the ratio between hemorrhagic and ischemic stroke aetiology is equal [30,31], with a slight AIS predominance (58.6% vs. 38.6% or 64% vs. 36%) [30,32].

## 4. Risk Factors

Pathogenesis of pediatric AIS seems to be different from adults in which atherosclerosis, diabetes mellitus, hypertension, smoking, metabolic syndrome, insulin resistance and chronic inflammatory conditions are well recognised risk factors [13]. In children AIS, the spectra of risk factors are even more comprehensive. However, for specific age groups, some factors seem to be more important than the others, according to Jeong et al. [33] (Table 1). In the perinatal period, pathogenesis is probably multifactorial (involving both maternal, neonatal and birth-related risk factors). The reported and presumed risk factors of perinatal stroke are infertility, primiparity, gestational hypertension, oligohydramnion, pre-eclampsia, chorioamnionitis, maternal fever, premature rupture of membranes, prolonged and instrumental/surgical delivery, birth asphyxia, trauma, early sepsis, cardiac disease, dehydration, hypercoagulable state (Factor V Leiden, Prothrombin G20210A mutation; Methylenetetrahydrofolate reductase mutation—MTHFR; Protein C or S deficiency; increased levels of factor VIII, IX, XI, fibrinogen, lipoprotein (a), hyperhomocysteinemia and antiphospholipid syndrome) and vasculopathies (predominantly artheriopathies) [13,14,16,34,35,36,37,38,39,40,41]. The identified risk factors for childhood AIS are artheriopaties, chronic systemic disease with inflammation, sickle cell anaemia, cardiac diseases and hypercoagulable states (thrombophilia), metabolic diseases, trauma, infection, dehydration and cancer [4]. Cardiac aetiology has been identified as one of the most prevalent risk factors in about 20–30% of cases [4,13,40,42], where the risk of AIS in children with congenital heart disease is 19-fold increased. Artheriopathies (intra- and extracranial) such as Moyamoya, craniocervical arterial dissection (CCAD), vasculitis and focal cerebral arteriopathy of childhood (FCA) can represent risk factors up to 29% of reported cases [25]. The impact of artheriopaties could be highlighted by the 40–80% incidence of arterial abnormalities found on vascular imaging in children with AIS [26,43]. Thrombophilia was identified in 20–50% of children with AIS (considerably higher incidence compared to adult AIS patients) [37,44]. Sickle cell disease (SCD) is a significant risk factor. It could be the most important regionally-based AIS risk in areas with a higher prevalence of SCD (e.g., in Sub-Saharan Africa, South Asia, the Middle East and the Mediterranean), with the peak incidence in children between 2 and 5 years [45,46]. A higher incidence of AIS is reported in the Black race and male gender [31,47]. Although in 50–80% of patients with AIS, at least one risk factor has been identified [4,48]. In 25% no risk factor could be identified and AIS has been classified as idiopathic [22,49]. Recently, during the global SARS-CoV-2 pandemic, there were several reported case scenarios of AIS occurring in children who either suffered acute respiratory distress syndrome caused by SARS-CoV-2 or who presented with AIS and other symptoms as part of the paediatric multisystem inflammatory response temporally associated with COVID-19 (PIMS-TS or multisystem inflammatory syndrome in children—MIS-C) [50,51]. There were concerns about the possible higher incidence of AIS in children, which might be explained by uncontrolled inflammatory response and cytokine storm following infection with SARS-CoV-2 and decreased mobility during the quarantine as a risk factor for venous thromboembolism. However, the data of children stroke cases positive for SARS-CoV-2 are insufficient [52].

## 5. Presentation and Diagnosis

Immediate AIS diagnosis in combination with imaging is the mainstay of the initial management. However, due to age-related differences in clinical presentation and even non-specific stroke symptoms in newborns, infants, toddlers and small children, the median time for AIS diagnosis in children is significantly longer compared to the adult population [53,54]. The reported interval from initial symptoms to hospital admission is highly variable, the delay to a definitive diagnosis of AIS reaches the median time between 15 and 24 h [13] and the in-hospital (admission to diagnosis) delay represents most of it [53]. When considering the perinatal stroke, the new-onset seizures, which typically include focal motoric unilateral seizures, are the most prevalent symptoms that occur in up to 94% of newborns (compared to 17–34% in childhood stroke) [13,29,55]. Non-specific cardiorespiratory syndromes are far more prevalent in newborns, whereas older children present with more typical symptoms: hemiparesis, hemifacial weakness, speech and vision abnormalities and altered consciousness [13]. Screening stroke pathways and stroke protocols implemented into emergency care could improve and speed up the diagnosis process. Several non-specific stroke-like conditions such as the new onset of a migraine, severe headache, Bell palsy and seizures with Todd paresis, brain tumour, central nervous infection, intoxication, traumatic brain injury, metabolic and/or psychiatric disease could mimic AIS (Table 2) [13]. The new onset of the focal deficit is more common in stroke patients than patients with stroke mimic presentation [13]. However, patients presenting with stroke-like symptoms should be transferred ideally to the specific stroke centre (e.g., paediatric stroke centre) with the possibility of 24/7 magnetic resonance imaging (MRI) and a paediatric neurologist on-site available to examine the patient initially in the emergency department before further advances. For the initial neurologic examination of a child with possible AIS, the National Institutes of Health Stroke Scale was validated for children between 2 and 17 years of age [56,57,58].

## 6. Initial Approach

**Figure 1:**
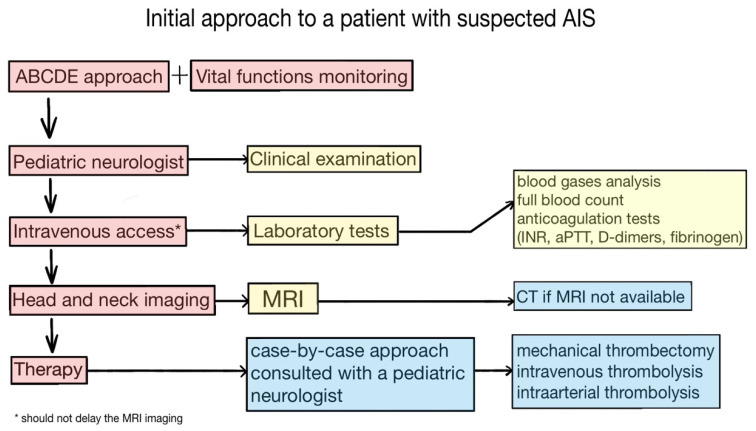
Standardized initial approach to a patient with suspected AIS.

Children with suspected AIS should be acutely transferred to the specialised pediatric stroke centres, where the stroke protocol/pathway should be initiated before the patient arrives at the emergency department. The mainstay of the good clinical practice could be considered as a standardised ABCDE approach implementation (according to the European Resuscitation Council—ERC; or European Paediatric Advanced Life Support—EPALS; Table 3) together with an acute neurologic examination (pediatric neurologist), intravenous access obtaining together with laboratory tests (Figure 1) and acute imaging method scheduling with the following primary aim: “Time is brain” (proceed as quickly as possible due to the possible time window for intravenous thrombolytic therapy and mechanical thrombectomy). The neurointensive care aimed for minimising the potential secondary damage by optimising the perfusion, oxygen delivery and even suppressing the oxygen radical formation (normal blood pressure for age, normal oxygen saturation, normocapnia, seizures treatment, normoglycemia and normothermia) should start immediately upon patient admission.

## 7. Imaging

In contrast to adult care where computed tomography (CT) remains the first imaging method, the MRI is considered a gold standard for children [4]. The initial CT scan in children could be falsely negative [56] (may miss AIS diagnosis in up to 50% of patient cases) [30] and can miss hyperacute small lesions or lesions located in the posterior fossa and brainstem [4]. Due to the risk of arteriopathy and dissection, the recommended approach is to perform an acute MRI of the head and neck. The optimal requirements for MRI stroke protocol are the following: diffusion-weighted imaging (DWI); magnetic resonance angiography (MRA); axial T2 fluid-attenuated inversion recovery (T2-FLAIR); susceptibility-weighted imaging or gradient echo (SWI or GRE) with approximately 25 min of MRI imaging duration or it could be limited to only DWI + SWI/GRE protocol to speed up the process [56]. In clinical practice, the significant delay in diagnosis could be affected by MRI availability and, in the case of infants, by the availability of an anaesthetist to provide general anaesthesia for imaging. In all children with AIS, echocardiography should be performed to rule out the possible cardio-embolic aetiology; however, this must not delay specific AIS therapy initiation (thrombolysis or thrombectomy).

## 8. Specific AIS Therapy

Based on adult data, intravenous, intraarterial thrombolysis (tissue plasminogen activator = tPA—Alteplase) and mechanical/endovascular thrombectomy could be considered for pediatric patients under 18 years with AIS [59]. The treatment efficacy is directly proportional to the delay (minimum delay = better outcome) and should be initiated in predefined time windows [60,61]. For intravenous tPA 4.5 h and for intra-arterial and mechanical thrombectomy 6 h from the onset of AIS symptomatology with the possible window prolongation up to 24 h (for mechanical thrombectomy) in selected cases (basilar artery and middle cerebral artery thrombosis) [62,63]. Due to insufficient data considering the ideal tPA dosing in pediatric patients (TIPS—Thrombolysis in Pediatric Stroke trial was prematurely stopped due to enrollment issues), the adult dosing regimen should be used (0.9 mg/kg, 10% of the total dose administered as i.v. bolus over 1 min and the remainder infused over 60 min) [64]. However, the standard implementation in these treatment regimens to pediatric patients should be still based on a case-by-case approach because 30–50% of patients with AIS could recover without neurologic deficits and without treatment [60,65] and the risk of intervention could possibly be higher than the benefit [13], in particular, in children with low risk predicted by the initial Pediatric NIH Stroke Scale [66,67]. According to recently published guidelines for stroke management in children and neonates (Ferriero et al.), the intervention should be considered in older children with NIH Stroke Scale ≥ 6 and proven large artery occlusion after neurologist and endovascular surgeon consultation [13]. Although the overall risk of symptomatic intracranial haemorrhage after intravenous tPA is low (around 2.6%) [68] and even lower in older children and young adults [69], the risk of intracranial haemorrhage is approximately 3.48 higher after tPA compared to no treatment with no effect on in-hospital mortality according to the recently published meta-analysis by Pacheco et al. [68]. Mechanical endovascular thrombectomy should be considered when available in children with basilar and middle cerebral artery occlusion due to thrombus/embolus formation. The method has highly reported in recanalisation rates, low procedure associated risks (when performed by trained endovascular surgeon/radiologist) and excellent clinical outcome (up to 87.6% by modified Rankin scale) [70]. Surgical hemicraniectomy should be considered as a potentially life-saving procedure in pediatric patients with large supratentorial ischemic areas and large cerebellar infarction (e.g., middle cerebral artery occlusion), where specific therapy (thrombolysis and thrombectomy) was not indicated or failed [13]. It can be performed as early prophylactic (in the first 24 h) or in 72 h (based on serial imaging) [13]. The overall reported survival rate after decompressive craniectomy series for pediatric AIS reached 95%, with 59% of patients with severe neurologic deficits [13]. Specific treatment is required in patients with AIS based on SCD aetiology, where exchange transfusion is urgent to improve the cerebral blood flow [71] and to reach haemoglobin levels up to 10 g/dL and lower the haemoglobin levels S ≤ 15% [13].

## 9. Further Treatment and Recurrence Prevention

Antithrombotic therapy (ATT) should be initiated in children with AIS as primary and secondary prevention. Aspirin or low molecular-weight heparin (LMWH) is recommended for initial treatment [72]. ATT in children with AIS appears to be safe in the initial treatment [73,74] and significantly reduces AIS recurrence risk [73,75]. In children with AIS based on cardiac aetiology, artheriopathy, extracranial dissection and prothrombotic disorder (e.g., thrombophilia), LMWH or even warfarin should be preferred for 3–6 months after stroke (even longer in selected cases based on haematologist recommendation) [13,72]. Aspirin (3–5 mg/kg/day) is recommended for prevention in all other AIS cases (dominantly in idiopathic AIS) [13]. Based on published data, ATT has been administered in the majority of childhood AIS (60%) but only in the minority of perinatal AIS (13%), probably due to lower risks of recurrence in perinatal AIS [30]. The risk associated with anticoagulation therapy (4% risk of symptomatic and 7% of asymptomatic intracranial haemorrhage) [73] should be compared with the risk of recurrence without ATT (1.5–2.0 risk) [29] in the following two years [76]. ATT (heparin) is contraindicated in the acute phase in AIS with hemorrhagic diathesis, bleeding disorders and even with the high stroke volume infarction with the highest risk for hemorrhagic transformation (e.g., complete middle cerebral artery occlusion) [56,77]. When considering the risk of potential administration of ATT, the ATT should be individualised and based on the consultation with a paediatric haematologist.

Further neurointensive care consists in primarily supportive therapy to maintain normoxemia, normocapnia, normotension (50–95th percentile age/height), normothermia, normoglycemia, euvolemia, aggressive seizure control (even continuous EEG implementation if indicated) and early rehabilitation [15,56,72,78]. In patients with intracranial hypertension and those possessing a risk of herniation, osmotic therapy with mannitol and/or hypertonic saline and decompressive craniectomy should be considered [13,78,79,80]. The benefit of intracranial pressure monitoring in patients with AIS is currently controversial, with inconsistent results [80,81].

## 10. Outcome

Reported pediatric AIS long-term outcomes are variable with significant (moderate to severe) neurologic deficit diagnosed in 31–51%, motor deficits between 50–62% and normal outcome (without deficit) in 30% patients [29,82,83,84,85]. The reported childhood AIS-related mortality is between 4 and 16% and has significantly decreased over time [29,30,86,87]. The risk of post-stroke epilepsy is around 25%, which is directly proportional to the volume of cortical ischemia [85,88]. The negative neurologic prognosis is strongly associated with initial NIH Stroke Scale [66,67], infarction volume [89] and imaging abnormalities (e.g., arteriopathies) [3,37,89].

## 11. Conclusions

Although new AIS risk factors, such as COVID-19 has been identified and the AIS incidence over the past decades is rising the overall AIS-related mortality is progressively decreasing The better access relative to imaging methods (dominantly MRI), pediatric stroke protocols implementation, the establishment of pediatric stroke centres, together with AIS specific therapy for high-risk patients and guidelines for pediatric stroke diagnosis and management could be a possible explanation. The mainstay of positive outcomes remains to be early AIS recognition, standardised approaches and early therapy (case-by-case approach) in high-risk patients.

## Figures and Tables

**Table 1:** AIS age-related risk factors.

Most Common Risk Factors According to Age Group (According to Jeong et al. [33])	
Age Group	Most Common Risk Factor
1–11 months	CNS infection
	Cardiac disease Severe dehydration
1–5 years	Moyamoya disease
	Cardiac disease
	Inflammatory vasculopathy
6–11 years	Moyamoya disease
	Prothrombotic condition
	Metabolic disease
≥12 years	Cardiac disease
	Prothrombotic condition
	Metabolic disease

**Table 2:** Possible clinical AIS presentation.

	Clinical AIS Presentation	
Perinatal AIS	Childhood AIS	Stroke-Like Symptoms
Seizures (focal and unilateral)	Hemiparesis	Migraine
Cardiorespiratory symptoms	Facial unilateral weakness	Headache
Altered consciousness	Speech disorder	Confusion
Failure to thrive	Vision abnormalities	Syncope
Feeding intolerance	Altered consciousness	Nausea and vomiting
		seizures with Todd paresis
		Bell palsy
		Altered consciousness

**Table 3:** Recommended initial approach to a patient at emergency.

ABCDE Approach by ERC and EPALS *		
ABCDE Approach	Aim	Action/Management
**A—Airway**	Airway patency, cervical spine protection if indicated	Open the mouth, bend the head (over 1 year), use airway if needed, MILS **, cervical collar or head blocks
**B—Breathing**	Spontaneous breathing efficacy, normoxemia, normocapnia	Pulse oximetry, oxygen, mechanical ventilation if indicated, capnography and blood gases analysis
**C—Circulation**	Oxygen delivery to meet the demand, blood pressure (50–95% according to age), adequate heart rate, capillary refill time ≤2 s, lactate ≤2 mmol/L	Fluid resuscitation (10 mL/kg fluid challenge), vasopressors or antihypertensives to meet target blood pressure
**D—Disability**	GCS ≥ 9, seizures control	Tracheal intubation and mechanical ventilation if GCS ≤ 8 and anticonvulsants
**E—Exposure/Examination**	Clinical examination, temperature management, normoglycemia (6–10 mmol/L)	Insulin or glucose to meet target glycemia and normothermia

* ERC (European Resuscitation Council); EPALS (European Paediatric Advanced Life Support). ** Manual in-line stabilisation (of the cervical spine). ABCDE—universal initial approach to the patient, considering the importance of vital signs in alphabetical order.
